# Is a universal nurse home visiting program possible? A cross-sectional survey of nurse home visitation service needs among pregnant women and mothers with young children

**DOI:** 10.1371/journal.pone.0272227

**Published:** 2022-08-04

**Authors:** Young-Ho Khang, Kyung Ja June, Sae Eun Park, Sung-Hyun Cho, Ji Yun Lee, Yu-Mi Kim, Hong-Jun Cho

**Affiliations:** 1 The Support Team for the Early Life Health Management Project, Seoul, Korea; 2 The Support Team for the Seoul Healthy First Step Project, Seoul, Korea; 3 Department of Health Policy and Management, Seoul National University College of Medicine, Seoul, Korea; 4 Department of Nursing, Soonchunhyang University, Cheonan, Korea; 5 Institute of Health Policy and Management, Seoul National University Medical Research Center, Seoul, Korea; 6 College of Nursing, Research Institute of Nursing Science, Seoul National University, Seoul, Korea; 7 College of Nursing, Kangwon National University, Chuncheon, Korea; 8 Department of Preventive Medicine, Hanyang University College of Medicine, Seoul, Korea; 9 Department of Family Medicine, Asan Medical Center, University of Ulsan College of Medicine, Seoul, Korea; Chinese University of Hong Kong, HONG KONG

## Abstract

In 2019, the South Korean government established a plan to develop home visitation services for pregnant women and women with children below the age of 24 months and expand the services nationwide. Therefore, a national survey was needed to provide relevant information for the policy decision of whether to implement universal home visitation services by nurses for families with young children. To determine home visitation service needs in South Korea, 804 women who were pregnant or had children below the age of 24 months were selected as survey participants through stratified random sampling by region reflecting geographical distribution in numbers of births. Of them, 614 responded to survey questionnaires delivered via email. After excluding surveys with too short of a response time, extreme values, and incomplete answers, 500 participants’ responses were analyzed. Participants indicated whether they supported the provision of home visitation services and whether they were willing to utilize home visitation services. The survey also elicited responses regarding the level of needs for individual service items that could be delivered by nurses during home visits. The fieldwork was conducted by a consulting and research firm. The differences in whether respondents supported nurse home visitation services and intended to use nurse home visitation services according to mothers’ characteristics were examined using the chi-square test. In total, 88.0% of survey participants supported nurse home visitation services, and 81.2% indicated that they intended to receive the services. Most pregnant women and women with children below the age of 24 months responded positively to the various prenatal or postpartum services that nurses could provide during home visits. The percentages of support for the services and intention to use services were generally high among subgroups according to mothers’ characteristics. Therefore, universal home visitation services by nurses during pregnancy and in the postnatal period would be received well by Korean women.

## Introduction

The prenatal period and early childhood are critical phases to promote health and well-being in adulthood [1,2]. To reduce health inequalities, interventions targeting the prenatal period and early childhood are necessary [3,4]. Such interventions have very high benefit-to-cost ratios [5–8], and studies have argued that societal investment in these periods has no equity-efficiency tradeoff [2,9]. Prenatal and early childhood home visitation services have been expanding, mainly in high-income countries, as a strategy to prevent exposure to risk factors adversely affecting children’s health and development and to strengthen protective factors within families [10–12].

In South Korea (hereafter, Korea), a prenatal and early childhood home visitation program was first implemented in 2013 through the Seoul Healthy First Step Project [13]. This program provides universal home visitation by nurses, multiple focused home visits, group activities, and community service linkage by social workers. In 2019, the Korean government established the Inclusive State Child Policy. This policy included a plan to develop home visitation services for women with young children below the age of 24 months and expand the services nationwide [14]. In preliminary drafts of the government’s plan, home visitation services were planned for families with a high risk of childhood maltreatment, including home visits for disadvantaged women who were pregnant or had children below the age of 24 months. However, universal home visitation was not mentioned explicitly in the preliminary drafts.

Along with multiple targeted home visitation services, several developed countries in Europe (including the Netherlands, Sweden, and United Kingdom) and Australasia (including Australia and New Zealand) have implemented universal home visitation (mainly provided by nurses) [10–12]. Meanwhile, targeted home visitation services for high-risk families are usually provided in the United States, even though that country does not have universal home visits [11,12]. Universal home visitation is considered the better option to identify families at a high risk of child abuse, who need regular and targeted services, because children from any socioeconomic background can experience health or developmental issues. It was reported that the Family Nurse Partnership program, which targets young low-income mothers, could only respond to around 10% of children with developmental issues [15]. A universal service infrastructure would make it possible to identify more families at a high risk of child abuse, but the question remains of whether mothers will “open their doors” to home visitors.

This study investigated home visitation service needs among pregnant women and mothers who had children below the age of 24 months. This survey was planned as part of a larger study to establish a home visitation program for pregnant women and infants, as an initiative of the Korean government [14]. The outcomes of this study could inform the planning and implementation of a nurse home visitation program that has been scheduled for nationwide expansion in Korea. This study is expected to provide relevant information for the policy decision of whether to implement universal home visitation services by nurses for families with young children in Korea.

## Methods

### Study participants

A written consent for each participant was obtained. The study was conducted according to the guidelines of the Declaration of Helsinki. The Institutional Review Board of Seoul National University Hospital approved the study (IRB No. 1908-115-1056).

The study population was pregnant women and mothers with children below the age of 24 months. The fieldwork was conducted by Macromill Embrain, a consulting and research firm. Among 1,310,000 people registered in the Macromill Embrain panel, 490,000 were women aged between 25 and 45. From this sample, 4,520 women were selected through stratified random sampling by region and contacted via email to identify those who wished to participate. The sample size per region (metropolitan city and province) was allocated based on the birth data by metropolitan city and province in 2017 published by Statistics Korea [16]. First, 804 women who met the eligibility criteria (either being pregnant or having a child below the age of 24 months) were selected as survey participants. The survey was distributed to these women, of whom 613 responded. Written informed consent for each participant was obtained. Surveys with too short of a response time, extreme values, and incomplete answers to open-ended questions were excluded. A final sample of 500 (150 pregnant women and 350 mothers with children below the age of 24 months) was included in the analysis.

### Questionnaires

The content of the questionnaire included whether respondents supported nurse home visitation services, whether they intended to use home visitation services provided by nurses, the types of home visitation services they needed during pregnancy, and the types of home visitation services they needed postpartum. The questionnaire also included items on mothers’ characteristics (age, household income, education, smoking status, alcohol drinking status, and level of depression). The level of depression was measured using the 10-item Edinburgh Depression Scale [17]. Major depression is indicated by a depression score of 13 points or higher [18]. The researchers composed a draft questionnaire, and eight education nurses in the Seoul Healthy First Step Project reviewed and revised the draft based on the services they had provided. English and Korean versions of the questionnaire used in this study are presented in the supplementary information (S1 and S2 Figs, respectively).

First, whether pregnant women and mothers with children below the age of 24 months supported home visitation services was investigated using the following question. “What would be your opinion on a nurse from a public health center visiting your home, free of charge, to provide services such as helping with delivery preparation for pregnant women, helping with postpartum health, childcare, and breastfeeding for women postpartum, and checking the baby’s development and providing counseling?” Respondents could then reply by selecting either “I would support a nurse visiting my house.” or “I would not support a nurse visiting my house”.

Next, participants were asked about their intention to use home visitation services, using the following question: “If it was possible for a nurse from the public health center to visit your house and provide the abovementioned home visitation program for free, would you intend to receive that service?” Respondents could choose between responses of ‘no intention at all,’ ‘no intention,’ ‘moderate,’ ‘willing,’ and ‘very willing.’ Responses of ‘willing’ or ‘very willing’ were classified as indicating an intention to use home visitation services.

The need for each service item that could be provided during nurses’ home visits was measured using the following question: “If you receive a home visit from a nurse during your pregnancy, what type of services would you like? For each item, if you would like that service, please answer ‘yes,’ and if not, ‘no.’ If you are currently pregnant, please consider what type of help you currently need. If you are currently raising a child, please think about when you were pregnant.” Specific types of services were listed. Mothers with a child below the age of 24 months were asked “If you receive a home visit from a nurse while your child is below the age of 24 months, what type of help would you like? For each item, if you would like that service, please answer ‘yes,’ and if not, ‘no.’ Specific types of services were listed.

### Statistical analysis

Mothers’ support of nurse home visitation services was analyzed according to their general characteristics, such as pregnancy status, region of residence, maternal age, number of children, household income, education, alcohol and tobacco use, and perinatal depression. Mothers who were pregnant and at the same time had children below the age of 24 months (N = 9) were categorized as pregnant women, rather than as women with children below the age of 24 months, as we presented results by mothers’ status on pregnancy. Intention to use home visitation services was presented as the percentage of responses of ‘no intention at all,’ ‘no intention,’ ‘moderate,’ ‘willing,’ and ‘very willing.’ Intention to use these services (defined as responses of ‘willing’ or ‘very willing’) was also analyzed as a dichotomous variable. The differences in whether respondents supported nurse home visitation services and intended to use nurse home visitation services according to mothers’ characteristics were evaluated using the chi-square test. The chi-square test was also used to test differences according to region (Seoul and others). These subgroup-specific proportions of support and intention may help identify any population subgroups with low support and intention, which may hinder the universal approach of expanding nurse home visitation services. The percentage of respondents who stated that they would want each type of service in home visits during pregnancy and postpartum was presented. All statistical analyses were conducted using SAS version 9.4 (SAS Institute, Cary, NC, USA).

## Results

Table 1 shows the characteristics of the study participants (N = 500) according to pregnancy status. Since the sample was distributed based on the 2017 data on the number of births by metropolitan city and province from Statistics Korea, the regional distribution of participants was similar to the distribution of the number of births by region in Korea. The majority of participants (N = 415, 83.0%) were in their 30s. Yearly household income ranged from below 20,000 USD (1 USD = 1,000 Korean won) to more than 100,000 USD, but more than half of participants had household incomes between 30,000 USD and 70,000 USD. Most participants (N = 365, 73.0%) were university graduates. Additionally, 2.7% (N = 4) of pregnant women stated that they drank two or more times per week, which was significantly lower than the corresponding percentage of 21.4% (N = 75) of mothers with children below the age of 24 months. A similar percentage (2.0%, N = 3) of pregnant women answered that they were current smokers, while this was the case for 2.6% (N = 9) of mothers with children below the age of 24 months. Finally, 20.7% (N = 31) of pregnant women and 29.1% (N = 102) of mothers with children below the age of 24 months had scores of 13 or above on the Edinburgh Depression Scale.

**Table 1:** Characteristics of study participants according to pregnancy status (N = 500).

	Pregnant women (N = 150), N (%)	Women with children below the age of 24 months (N = 350), N (%)	Total study participants (N = 500), N (%)
Regions			
Seoul	28 (18.7)	64 (18.3)	92 (18.4)
Gyeonggi-do/Gangwon-do	52 (34.7)	120 (34.3)	172 (34.4)
Daejeon/Chungcheong-do	17 (11.3)	41 (11.7)	58 (11.6)
Gwangju/Jeolla-do/Jeju-do	16 (10.7)	38 (10.9)	54 (10.8)
Daegu/Gyeongsangbuk-do	14 (9.3)	33 (9.4)	47 (9.4)
Busan/Gyeongsangnam-do	23 (15.3)	54 (15.4)	77 (15.4)
Women’s age (years)			
20–29	18 (12.0)	40 (11.4)	58 (11.6)
30–39	122 (81.3)	293 (83.7)	415 (83.0)
40–49	10 (6.7)	17 (4.9)	27 (5.4)
No. of children (including fetus)			
1	104 (69.3)	193 (55.2)	297 (59.4)
2	41 (27.3)	129 (36.9)	170 (34.0)
3 or more	5 (3.3)	28 (8.0)	33 (6.6)
Annual household income (USD)			
Less than 20,000	3 (2.0)	17 (4.8)	20 (4.0)
20,000–29,999	14 (9.3)	34 (9.7)	48 (9.6)
30,000–49,999	48 (32.0)	123 (35.1)	171 (34.2)
50,000–69,999	47 (31.3)	107 (30.6)	154 (30.8)
70,000–999,999	34 (22.7)	52 (14.9)	86 (17.2)
100,000 or over	4 (2.7)	17 (4.9)	21 (4.2)
Educational attainment			
High school or less	15 (10.0)	27 (7.7)	42 (8.4)
College	30 (20.0)	63 (18.0)	93 (18.6)
University or more	105 (70.0)	260 (74.3)	365 (73.0)
Alcohol drinking, 2+ times per week			
Yes	4 (2.7)	75 (21.4)	79 (15.8)
No	146 (97.3)	275 (78.6)	421 (84.2)
Cigarette smoking			
No experience of smoking	116 (77.3)	293 (83.7)	409 (81.8)
Currently smoking	3 (2.0)	9 (2.6)	12 (2.4)
Ex-smokers	31 (20.7)	48 (13.7)	79 (15.8)
Edinburgh Depression Scale			
Less than 13	119 (79.3)	248 (70.9)	367 (73.4)
13 or over	31 (20.7)	102 (29.1)	133 (26.6)

Note: 1 USD = 1,000 Korean won.

Table 2 presents the proportions of respondents who supported nurse home visitation services. The vast majority (88%, N = 440) of both pregnant women and mothers with children below the age of 24 months supported nurse home visitation services. The lowest proportion of support for nurse home visitation services was 75.8% among women with 3 or more children. The proportion of participants who supported these services was highest in Seoul, but lowest in Daejeon/Chungcheong-do. The proportion of support was 92.4% in Seoul and 87.0% in other regions (p = 0.118). Younger age, higher education levels, and higher income tended to be associated with a higher proportion of support for nurse visitation services, but these trends were not statistically significant. A slightly lower rate of support was found among respondents with 3 or more children (75.8%), and this difference was statistically significant (p = 0.041). The presence of depression showed no statistically significant relationship with participants’ intention to use services (p = 0.525).

**Table 2:** Support for nurse home visitation services (%) among pregnant women and mothers with children below the age of 24 months (N = 500).

Characteristics	No. of subjects (%)	Support for nurse home visitation services (%)	P value
Total study participants	500 (100.0)	88.0	NA
Pregnancy status			
Pregnant women	150 (30.0)	88.0	1.000
Women with children below the age of 24 months	350 (70.0)	88.0	
Regions			
Seoul	92 (18.4)	92.4	0.151*
Gyeonggi-do/Gangwon-do	172 (34.4)	86.6	
Daejeon/Chungcheong-do	58 (11.6)	84.5	
Gwangju/Jeolla-do/Jeju-do	54 (10.8)	85.2	
Daegu/Gyeongsangbuk-do	47 (9.4)	87.2	
Busan/Gyeongsangnam-do	77 (15.4)	90.9	
Women’s age (years)			
20–29	58 (11.6)	93.1	0.279
30–39	415 (83.0)	87.7	
40–49	27 (5.4)	81.5	
No. of children (including fetus)			
1	297 (59.4)	87.5	0.041
2	170 (34.0)	91.2	
3 or more	33 (6.6)	75.8	
Annual household income (USD)			
Less than 20,000	20 (4.0)	80.0	0.743
20,000–29,999	48 (9.6)	85.4	
30,000–49,999	171 (34.2)	88.9	
50,000–69,999	154 (30.8)	87.7	
70,000–999,999	86 (17.2)	88.4	
100,000 or over	21 (4.2)	95.2	
Educational attainment			
High school or less	42 (8.4)	81.0	0.160
College	93 (18.6)	85.0	
University or more	365 (73.0)	91.6	
Alcohol drinking, 2+ times per week			
Yes	79 (15.8)	84.8	0.342
No	421 (84.2)	88.6	
Cigarette smoking			
No experience of smoking	409 (81.8)	87.5	0.493
Currently smoking	12 (2.4)	75.0	
Ex-smokers	79 (15.8)	92.4	
Edinburgh Depression Scale			
Less than 13	367 (73.4)	88.6	0.525
13 or over	133 (26.6)	86.5	

Note: 1 USD = 1,000 Korean won. NA = not available.

*The chi-square test was performed to calculate the statistical significance of differences between Seoul and other regions.

Table 3 presents the intention to use nurse home visitation services among pregnant women and mothers with children below the age of 24 months in Korea. Most respondents (81.2%, N = 406) were willing to or very willing to receive home visitation services provided by nurses, as 82.7% of pregnant women and 80.6% of mothers with children below the age of 24 months responded that they were either ‘willing’ or ‘very willing.’ If the ‘moderate’ response was included as a positive answer, 94.0% (95.4% of pregnant women and 93.6% of mothers with children below the age of 24 months) of participants expressed an intention to use home visitation services. Only 1% (n = 5) of respondents strongly refused the services by answering ‘not willing at all.’ With the exception of women with 3 or more children, fewer than 10% of participants responded that they had either ‘no intention at all’ or ‘no intention’.

**Table 3:** Intention to use nurse home visitation services (%) among pregnant women and mothers with children below the age of 24 months (N = 500).

Characteristics	No intention at all (%)	No intention (%)	Moderate (%)	Willing (%)	Very willing (%)	Willing or very willing (%)	P value
Total study participants	1.0	5.0	12.8	44.2	37.0	81.2	NA
Pregnancy status							
Pregnant women	1.3	3.3	12.7	42.7	40.0	82.7	0.583
Women with children below the age of 24 months	0.9	5.7	12.9	44.9	35.7	80.6	
Regions							
Seoul	0	2.2	10.9	42.4	44.6	87.0	0.118*
Gyeonggi-do/Gangwon-do	1.7	7.6	13.4	44.8	32.6	77.3	
Daejeon/Chungcheong-do	1.7	3.5	17.2	41.4	36.2	77.6	
Gwangju/Jeolla-do/Jeju-do	0.0	5.6	11.1	40.7	42.6	83.3	
Daegu/Gyeongsangbuk-do	0.0	4.3	14.9	48.9	31.9	80.9	
Busan/Gyeongsangnam-do	1.3	3.9	10.4	46.8	37.7	84.4	
Women’s age (years)							
20–29	1.7	3.5	10.3	32.8	51.7	84.5	0.286
30–39	0.7	5.3	12.5	45.3	36.1	81.5	
40–49	3.7	3.7	22.2	51.9	18.5	70.4	
No. of children (including fetus)							
1	1.0	4.4	11.8	46.1	36.7	82.8	0.235
2	1.2	4.7	13.5	41.2	39.4	80.6	
3 or more	0.0	12.1	18.2	42.4	27.3	69.7	
Annual household income (USD)							
Less than 20,000	0.0	0.0	35.0	35.0	30.0	65.0	0.162
20,000–29,999	2.1	6.3	16.7	43.8	31.3	75.0	
30,000–49,999	0.0	5.3	9.9	46.2	38.6	84.8	
50,000–69,999	1.3	5.8	13.0	40.3	39.6	79.9	
70,000–999,999	2.3	4.7	14.0	48.8	30.2	79.1	
100,000 or over	0.0	0.0	0.0	47.6	52.4	100.0	
Educational attainment							
High school or less	2.4	7.1	14.3	31	45.2	76.2	0.009
College	1.1	6.5	21.5	40.9	30.1	71.0	
University or more	1.3	2.6	9.0	49.1	38.2	83.1	
Alcohol drinking, 2+ times per week							
Yes	0.0	8.9	16.5	44.3	30.4	74.7	0.106
No	1.2	4.3	12.1	44.2	38.2	82.4	
Cigarette smoking							
No experience of smoking	1.2	5.4	12	44.7	36.7	81.4	0.791
Currently smoking	0.0	0.0	33.3	50.0	16.7	66.7	
Ex-smokers	0.0	3.8	13.9	40.5	41.8	82.3	
Edinburgh Depression Scale							
Less than 13	0.5	4.9	13.1	46.1	35.4	81.5	0.796
13 or over	2.3	5.3	12	39.1	41.4	80.5	

Note: 1 USD = 1,000 Korean won. NA = not available.

*The chi-square test was performed to determine the statistical significance of differences between Seoul and other regions.

Intention to use these services was highest in Seoul, but lowest in Gyeonggi-do/Gangwon-do. The percentage of participants who intended to use these services was non-significantly higher in Seoul (87.0%) than in other regions (79.0%) (p = 0.118). Similarly, statistically non-significant associations with positive intention to use nurse home visitation services were found among younger mothers, those with fewer children, and those with higher incomes. Higher education levels were significantly associated with intention to use these services (p = 0.009), and no significant relationship was found for depression (p = 0.796).

Table 4 displays the need for prenatal nurse home visit services for each item. The most common service needs were for diarrhea, vomiting, and fever in newborn infants (97.4%), followed by baby sleep (96.2%), belly button and skin condition (95.4%), and home environment for the baby (95.4%). High levels of educational needs for husbands and partners were reported for infant care by the husband or partner (96.6%), understanding pregnancy and childbirth (95.6%), and supporting the mother during labor (95.2%). Among the services related to self-care during pregnancy, high levels of need were expressed for nutrition and physical activity during pregnancy (91.6%) (Table 4).

**Table 4:** Needs (%) for prenatal nurse home visitation services among pregnant women and women with children below the age of 24 months (N = 500).

Service items	%	Service items	%
Information related to childbirth		Feeding the baby	
Signs of labor	91.0	Overall information on feeding the baby	92.4
Process of labor and breathing methods	85.2	Preparation for breastfeeding	92.4
Maternal and fetal changes by trimesters	82.2	Psychological support	
Self-care during pregnancy		Understanding mood changes	89.6
Nutrition and physical activity during pregnancy	91.6	Stress management during pregnancy	89.0
Perineum care during pregnancy	85.0	Screening of prenatal depression	87.8
Dental health during pregnancy	84.0	Maternal and fetal interaction	
Constipation issues during pregnancy	81.4	Prenatal education	89.6
Understanding babies and baby care		Husband and partner education	
Diarrhea, vomiting, and fever in newborn infants	97.4	Infant care by husband or partner	96.6
Baby sleep	96.2	Understanding pregnancy and childbirth	95.6
Belly button and skin condition	95.4	Supporting the mother during labor	95.2
Infant growth and development	95.2	Contraception	75.2
Prevention of sudden infant death syndrome	95.2	Home environment	
Infant’s normal body temperature	95.0	Home environment for the baby	95.4
Baby massage	94.8	Home safety	94.0
Assessing baby’s urine output and feces	93.8	Indoor temperature for the infant	90.4
Infant crying	93.8		
Baby bath	91.8		

Table 5 presents the needs for postnatal nurse home visit services by service items. High levels of service needs were found for physical examination of the baby (98.9%), growth evaluation (98.6%), belly button and skin conditions (97.7%), dental health of babies (96.6%), and communication with the baby (95.4%) (Table 5). Among the needs related to education for husbands and partners, high levels of service needs were found for massage for the mother (94.0%), emotional support for depressed mothers (93.7%), and husbands’ baby care (e.g., diaper changing) (90.0%). Of the items related to the mother’s self-care, the highest service need was found for postnatal gymnastic exercises (91.4%) (Table 5).

**Table 5:** Needs (%) for postnatal nurse home visitation services among pregnant women and women with children below the age of 24 months (N = 500).

Service items	%	Service items	%
Infant feeding		Baby care	
Amount and frequency of infant feeding	80.0	Dental health of babies	96.6
Breastfeeding and bottle-feeding methods	74.3	Baby play and providing toys	94.9
Burping a newborn after feeding	65.4	Baby massage	93.7
Maternal self-care		Vaccination and health examination	90.6
Postnatal gymnastic exercises	91.4	Infant crying	88.3
Nutrition and physical activity	88.0	Soothing baby to sleep	87.1
Mastitis prevention and treatment	84.3	Weaning food	84.0
Body weight management	81.4	Baby bath	82.0
Breast massage	80.9	Diaper change	61.4
Lochia assessment and care	72.6	Education for husbands or partners	
Urinary incontinence care	62.3	Massage for the mother	94.0
Contraception	55.1	Emotional support for the depressed mother	93.7
Psychological support		Husbands’ baby care (e.g., diaper changing)	90.0
Emotional support	89.1	Baby bath by partners	87.4
Screening of postnatal depression	87.1	Contraception	76.0
Anxiety issues	85.7	Parenting and home environment	
Mothers’ aspirations		Addressing health emergencies	98.9
Aspirations for the child’s future	82.6	Home safety	91.1
Aspirations for one’s future self	79.1	Use of a car seat for infants	69.4
Career consultations	73.4	Financial issues	48.9
Understanding babies		Housing issues	46.0
Baby physical examination	98.9	Other family members	
Growth evaluation	98.6	Relationship with older siblings	85.7
Belly button and skin conditions	97.7	Health issues of siblings or partner	81.1
Communication with the baby	95.4	Relationship with extended family members	57.4
Sudden infant death syndrome	93.4		
Assessing infant’s urine output and feces	92.3		

## Discussion

Targeted and regular prenatal and early childhood home visitation services for high-risk disadvantaged populations have a high benefit-to-cost ratio as an intervention to reduce health inequalities [5–8]. Randomized controlled trials on universal postnatal nurse home visiting services have also reported promising results for decreases in emergency care and child maltreatment [19,20]. These randomized controlled trials also indicated, based on an examination of billing records of emergency care [19,20], a $3.17 decrease in total billing costs for each $1 in costs for a universal nurse home visiting program [20]. Universal nurse home visitation services might also minimize stigmatization, which often arises with approaches targeting disadvantaged groups in a community, thereby contributing to an increase in program engagement among the eligible population. In European nations (including the United Kingdom) and Australia, focused and regular home visitation services for high-risk mothers based on universal home visitation by nurses have been provided [11,12,21]. The Korean central government established a policy to introduce nurse home visitation services nationally in 2019 [14], necessitating a decision on whether to include universal service components. In Korea, nationally representative studies have been conducted on fertility [22] and postpartum care [23]. However, the data regarding prenatal and postpartum home visitation services in Korea are limited, hindering the understanding of mothers’ needs for new home visitation services. A small local study in Korea measured home visitation service needs among families with preschool-age children, but those children were on average older than 30 months [24]. In contrast, this study aimed to understand needs for nurse home visitation services among pregnant women and mothers with children below the age of 24 months, who are the target population of the Korean central government’s initiative for the national expansion of home visitation services.

Most participants (88.0%) in this study supported nurse home visitation services. The lowest proportion of support was 75.8%, among women with 3 or more children (Table 2). In addition, the intention to use these services was also high (81.2%). A total of 82.7% of pregnant women and 80.6% of mothers with children below the age of 24 months responded that they either were ‘willing’ or ‘very willing’ to receive the services. If ‘moderate’ responses were included as a positive answer, 94.0% (95.4% of pregnant women and 93.6% of mothers with infants) intended to use home visitation services. In almost all sociodemographic subgroups, except for women with 3 or more children, fewer than 10% of participants indicated no intention to use home visiting services (Table 3). These findings indicate that a universal home visiting service is possible in Korea. Furthermore, the high level of support for universal nurse home visitation services found in this study could contribute to a policy decision of the Korean government to introduce universal home visiting.

In Korea, an ongoing project to provide nurse home visitation services during the prenatal period and early childhood started in Seoul in 2013. However, the service was not provided in other regions at the time of this study; therefore, the need to expand this program was raised [13]. In fact, the level of satisfaction with the universal visitation services in the Seoul Healthy First Step Project has been very high [13]. The high levels of satisfaction with the services support the possibility of universal services. Although the Korean government established a plan to nationally expand home visitation services for women with young children below the age of 24 months [14], that plan did not explicitly mention universal nurse home visitation. Along with the experience in Seoul indicating the plausibility of universal home visiting, the results of this study suggest that universal home visitation services could be well received by Korean pregnant women and women with children below the age of 24 months.

Internationally, examples of universal home visitation services can be found in many countries [10–12]. In Australia, the proportionate universality approach for families with infants has been implemented [25]. An Australian study reported that 82.1% of parents with young children received postpartum home visitation services from a child and family health nurse [25]. The prenatal and early childhood program in the Health Child Programme of the United Kingdom is based on the principle of progressive universal services that apply from pregnancy to when the child reaches the age of 5 [26]. The provision of universal services serves as a valuable opportunity to identify families that need extra support and children at risk for adverse outcomes. Along with our finding that Korean women had high levels of service needs and intention to use services, the successful experiences of universal home visitation services in Western countries with individualistic cultures stressing independence, self-reliance, and autonomy could serve as a good example for Korean policy makers to introduce universal home visitation services throughout the nation.

A previous study using British birth cohort data showed that only a small proportion of children with poor development can be identified using the mother’s age at delivery [27]. Meanwhile, when additional variables such as mothers’ education, financial difficulties, partner status, smoking, and depression are considered, a sizable number of children with developmental difficulties can be identified [27]. In other studies, additional information beyond the mother’s age and socioeconomic indicators improved the prediction of delayed child development in the future [28,29]. These variables can be obtained through universal prenatal evaluations and universal postpartum home visitation. Targeted approaches relying on limited information (usually mother’s age and socioeconomic indicators) from governmental administrative data might not be successful in addressing most developmentally vulnerable children in a community. In contrast, universal approaches with prenatal and postnatal universal evaluations of the maternal psychosocial environment and other detailed indicators could be better for approaching children at risk of developmental delay.

The high need for home visitation services found in this study is consistent with the needs identified among mothers in other Asian countries such as China and Singapore with less individualistic (i.e., more collectivist) cultures than in Western countries [30,31]. Like mothers in Korea, mothers in Singapore had a short period of hospitalization after delivery, and there were no services for mothers before the postpartum check-up. In that situation, mothers simultaneously felt complex emotions of happiness and negative feelings and were worried [30]. Mothers also wanted home visits from health practitioners for guidance and support regarding breastfeeding and childcare skills [31]. Collectivist cultures and underdeveloped perinatal community services might be reasons for the high levels of service needs in Asian countries.

This study showed that support for nurse home visitation services and the intention to use those services were not statistically significantly associated with mothers’ characteristics, excluding education level and number of children (including the fetus). High proportions of support and intention to use were found in all regions, but regions other than Seoul (i.e., in regions where the services have not been provided yet) tended to show slightly lower levels of intention to use. Supplementary Table (S1 Table) indicates that women in Seoul were more educated and had a relatively greater proportion of high annual household income than their counterparts in other regions outside of Seoul. Since education and household income were positively associated with the intention to use services (Table 2), these socioeconomic characteristics among women in Seoul might have partly contributed to the higher proportion of intention to use services in Seoul than elsewhere. In addition, S1 Table also shows that this tendency for higher levels of intention to use home visitation services among participants living in Seoul was consistent in each subgroup of survey participants. For example, the proportions of intention to use services according to women’s age, annual household income, alcohol drinking, and Edinburgh Depression Scale were all greater in Seoul than in other regions. A possible explanation for this finding is that these services are not widely known or familiar in regions outside of Seoul. This survey was conducted in 2019, when universal home visitation services were only available in Seoul. The findings of a slightly higher proportion of support and intention to use are presumed to result—at least partially—from the Seoul Healthy Frist Step Project.

The results of this study indicated a slightly lower intention to use home visitation services among mothers who consumed alcohol or tobacco and mothers who had low income levels. These mothers might have perceived services provided by public institutions as surveillance, had low expectations of service content, or had different attitudes toward parenting and parents’ role. This tendency underscores the need to provide home visitation services to all families with pregnant women and infants since the provision of universal services can become an opportunity to identify vulnerable families [32].

All proposed items for specific services needed in nurses’ home visits during pregnancy and postpartum had positive response rates of at least 73.2%, demonstrating high levels of need. The most frequently identified needs were related to understanding the baby’s condition and caring for the baby. Nurses, who are health practitioners, are expected to be able to help with these items, similar to how midwives and public health nurses were preferred by mothers as personnel for providing home visitation services in a study conducted in Japan [33].

The items with high service needs are also similar to those identified in the study with Japanese mothers [33] and overlap with the content of the universal visitation services provided by Child and Family Health Nurses in Australia [34] and child health nurses in the Seoul Healthy First Step Project [13]. The nurses who conduct the visits should be trained to provide education on infant physical examinations and growth evaluation, which were the specific items with the highest needs [35]. Moreover, the service needs for infant vaccination and health examinations can reasonably be met by linkage to existing child health services provided in local health clinics and public health centers.

Furthermore, active social support for postpartum depression is necessary, as a national study on fertility found that only 3.4% of mothers received professional help for postpartum depression in Korea [22]. The importance of such support is emphasized by the findings of this study that 23.0% of women in this study scored more than 13 points in the Edinburgh Depression Scale and 93.7% indicated service needs for husbands and partners’ education on providing emotional support for their depressive symptoms.

A limitation of this study is that the study participants may not have been nationally representative because they were sampled from a research firm panel. However, we tried to sample study participants through stratified sampling reflecting the regional distribution of childbirths in 2017. Therefore, we included participants from all metropolitan cities and provinces in Korea.

## Conclusion

Most pregnant women and mothers with children below the age of 24 months in Korea wanted nurse home visitation services. The study results indicate that universal home visitation services during pregnancy and after delivery could be received well by Korean women. When home visitation services become established as a universal service, universal home visits would provide an opportunity to identify vulnerable families that require focused services. The specific services found to be needed in this study could be integrated into the educational program and workflow planning for nurses who would provide home visitation services in Korea.

## Supporting information

S1 Fig Questionnaire for the survey of nurse home visitation service needs among pregnant women and mothers with young children below the age of 24 months (English version). (PDF)

S2 Fig Questionnaire for the survey of nurse home visitation service needs among pregnant women and mothers with young children below the age of 24 months (Korean version). (PDF)

S1 Table Intention to use nurse home visitation services among pregnant women and mothers with children below the age of 24 months in Seoul and other regions of Korea. (PDF)
