# Supplementary material for: Is a universal nurse home visiting program possible? A cross-sectional survey of nurse home visitation service needs among pregnant women and mothers with young children
Source: PLoS One. 2022 Aug 4;17(8):e0272227. doi: 10.1371/journal.pone.0272227 (PMC9352077; doi:10.1371/journal.pone.0272227)
Supplement: S1 Fig — (PDF) [file pone.0272227.s001.pdf]

S1 Fig. Questionnaire for the survey of nurse home visitation service needs among pregnant women and mothers with children below the age of 24 months (English version)

[Eligibility check for survey participation]

SQ1. What is your sex? 1) male 2) female

SQ2. What is your age? Year of birth: _________

SQ3. Which region do you reside in? (select a metropolitan city/province in the map.)

SQ4. How many children do you have? (including current pregnancies)

1) 5 2) 4 3) 3 4) 2 5) 1 6) 0

SQ4-1. Are you currently pregnant?

1) Yes 2) No

SQ4-2. How old are your children?

First child:	XX year XX months	Second child:	XX year XX months
Third child:	XX year XX months	Fourth child:	XX year XX months
Fifth child:	XX year XX months		

Hello,

This survey is being conducted as a part of our study on A Plan for the National Expansion of Prenatal and Early Childhood Home Visitation Service (Principal Investigator: Professor Young-Ho Khang, Seoul National University College of Medicine).

The results of this survey will be analyzed statistically to develop a nurse home visitation service for mothers and babies. No disadvantages result from not participating in this survey or discontinuing participation during the survey.

The estimated amount of time required for the survey is between 10 to 20 minutes. If you have a question about the survey, please use the help button on the bottom.

Individual responses will not be revealed, and all personal information will be protected. This survey will only be administered to participants who voluntarily participate.

□ Proceed to the survey □ Decline participation

[Common items for pregnant women and mothers with children aged less than 24 months]

1. What would be your opinion on a nurse from a public health center visiting your home, free of charge, to provide services such as helping with delivery preparation for pregnant women, helping with postpartum health, childcare, and breastfeeding for women postpartum, and checking the baby's development and providing counseling?

□ I would support a nurse visiting my home.

□ I would not support a nurse visiting my home.

2. If it was possible for a nurse from the public health center to visit your home and provide the abovementioned home visitation program for free, would you intend to receive that service?

□ No intention at all.

□ No intention.

□ Moderate.

□ Willing.

Please mark the answer which comes closest to how you have felt in the past 7 days, not just how you feel today.

3. I have been able to laugh and see the funny side of things:

□ As much as I always could

□ Not quite so much now

□ Definitely not so much now

□ Not at all

4. I have looked forward with enjoyment to things:

□ As much as I ever did

□ Rather less than I used to

□ Definitely less than I used to

□ Hardly at all

5. I have blamed myself unnecessarily when things went wrong:

□ Yes, most of the time

□ Yes, some of the time

□ Not very often

□ No, never

6. I have been anxious or worried for no good reason:

□ No, not at all

□ Hardly ever

□ Yes, sometimes

□ Yes, very often

7. I have felt scared or panicky for no good reason:

□ Yes, quite a lot

□ Yes, sometimes

□ No, not much

□ No, not at all

8. Things have been getting to me:

□ Yes, most of the time I haven't been able to cope at all

□ Yes, sometimes I haven't been coping as well as usual

□ No, most of the time I have coped quite well

□ No, I have been coping as well as ever

9. I have been so unhappy that I have had difficulty sleeping:

□ Yes, most of the time

□ Yes, sometimes

□ No, not very often

□ No, not at all

10. I have felt sad or miserable:

□ Yes, most of the time

□ Yes, quite often

□ Not very often

□ No, not at all

11. I have been so unhappy that I have been crying:

□ Yes, most of the time

□ Yes, quite often

□ Only occasionally

□ No, never

12. The thought of harming myself has occurred to me:

□ Yes, quite often

□ Sometimes

□ Hardly ever

□ Never

If you receive a home visit from a nurse during your pregnancy, what type of services would you like? For each item, if you would like that service, please answer “yes,” and if not, “no.” If you are currently pregnant, please consider what type of help you currently need. If you are currently raising a child, please think about what you needed when you were pregnant.

13-1. Help for mothers

Service item	Detailed description of service item	Yes	No
Information related to childbirth	Process of labor and breathing methods		
	Preparation to become a mother		
	Maternal and fetal changes by trimesters		
	Signs of labor		
Self-care during pregnancy	Nutrition and physical activity during pregnancy		
	Constipation issues during pregnancy		
	Perineum care during pregnancy		
	Dental health during pregnancy		
Psychological support	Understanding mood changes		
	Stress management during pregnancy		
	Screening of prenatal depression		
Maternal and fetal interaction	Prenatal education		
Feeding the baby	Overall information on feeding the baby		
	Preparation for breastfeeding		

13-2. Help needed in caring for the baby after delivery

Service item	Detailed description of service item	Yes	No
Infant growth and development	Infant growth and development		
Understanding babies	Prevention of sudden infant death syndrome		
	Infant's normal body temperature		
	Belly button and skin condition		
	Assessing baby's urine output and feces		
	Diarrhea, vomiting, and fever in newborn infants		
Baby care	Baby massage		
	Baby sleep		
	Baby bath		
	Infant crying		
	Diaper change		
	Temperature check		
	Skin rash check		
	Feeding amount		

13-3. Help needed from spouses/partners

Service item	Detailed description of service item	Yes	No
Husband and partner education	Understanding pregnancy and childbirth		
	Supporting the mother during labor		
	Infant care by husband or partner		
	Contraception		

13-4. Help needed regarding the home environment

Service item	Detailed description of service item	Yes	No
Home environment	Indoor temperature for the infant		
	Home safety		
	Home environment for the baby		

13-5. Please describe services other than those listed above that you need for your current pregnancy or needed when you were pregnant.

[Mothers with children aged less than 24 months]

Please answer the survey with information about the child to whom you most recently gave birth. Thank you.

For each statement think about how you have been feeling over the past 2–3 weeks.

Over the past 2–3 weeks

1. I have felt confident about looking after my baby/toddler

□ Yes, most or all of the time

□ Yes, some of the time

□ No, not very often

□ No, rarely or never

2. I have missed the life I had before I became pregnant with this baby/toddler (or for adoptive mothers: before I had this baby/toddler)

□ No, rarely or never

□ No, not very often

□ Yes, some of the time

□ Yes, most or all of the time

3. I have found it hard to cope when my baby/toddler cries

□ No, rarely or never

□ No, not very often

□ Yes, some of the time

□ Yes, most or all of the time

4. I have felt close to my baby/toddler

□ Yes, most or all of the time

□ Yes, some of the time

□ No, not very often

□ No, rarely or never

5. I have felt lonely or isolated

□ No, rarely or never

□ No, not very often

□ Yes, some of the time

□ Yes, most or all of the time

6. I have felt bored

□ No, rarely or never

□ No, not very often

□ Yes, some of the time

□ Yes, most or all of the time

7. I have felt unsupported

□ No, rarely or never

□ No, not very often

□ Yes, some of the time

Yes, most or all of the time

8. I have felt alright about asking people for help or advice when I needed to

□ Yes, most or all of the time

□ Yes, some of the time

□ No, not very often

□ No, rarely or never

9. I have felt nervous or uneasy around my baby/toddler

□ No, rarely or never

□ No, not very often

□ Yes, some of the time

□ Yes, most or all of the time

10. I have been worried that something would happen to my baby/toddler

□ No, rarely or never

□ No, not very often

□ Yes, some of the time

□ Yes, most or all of the time

11. I have been annoyed or irritated with my baby/toddler

□ No, rarely or never

□ No, not very often

□ Yes, some of the time

□ Yes, most or all of the time

12. I worry I am not as good as other mothers

□ No, rarely or never

□ No, not very often

□ Yes, some of the time

□ Yes, most or all of the time

13. I have felt guilty

□ No, rarely or never

□ No, not very often

□ Yes, some of the time

□ Yes, most or all of the time

14. During which period did you feel it was most difficult to care for your child?

□ Infancy (0-4 weeks)

□ Two to three months old (5-12 weeks)

□ Four to six months old (13-24 weeks)

□ Seven to 12 months old

□ 13 to 24 months old

15. Why was it difficult to care for your child during this period? (can select more than one)

□ Physical fatigue

□ Difficulty breastfeeding

□ Difficulty knowing what the baby wanted

□ Difficulty soothing the baby's crying

□ Depression

□ Isolation

□ Spouse or partner not helping

□ No support from others

□ Economic hardship

□ Worry about career break

□ Others (   )

The following items are services that a nurse can provide during a home visit after delivery and before the child reaches 24 months of age. If you were to receive a home visit from a nurse in this period, what type of help would you like? For each item, if you would like that service, please answer “yes,” and if not, “no.”

16-1. Help for mothers

Service item	Detailed description of service item	Yes	No
Infant feeding	Overall information on feeding the baby		
	Breastfeeding and bottle-feeding methods		
	Amount and frequency of infant feeding		
	Burping a newborn after feeding		
Maternal self-care	Breast massage		
	Mastitis prevention and treatment		
	Urinary incontinence care		
	Body weight management		
	Postnatal gymnastic exercises		
Self-care	Nutrition and physical activity		
	Lochia assessment and care		
	Contraception		
Child care skills	Responding to the baby		
	Understanding discipline		
Psychological support	Anxiety issues		
	Emotional support		
	Screening of postnatal depression		
	Career consultations		
Mothers' aspirations	Aspirations for one’s future self		
	Aspirations for the child’s future		

16-2. Help needed in caring for the baby

Service item	Detailed description of service item	Yes	No
Understanding babies	Growth evaluation		
	Sudden infant death syndrome		
	Baby physical examination		
	Belly button and skin conditions		
	Assessing infant's urine output and feces		
Baby care	Interacting with the baby		
	Baby massage		
	Baby bath		
	Infant crying		
	Soothing baby to sleep		
	Trimming nails		
	Diaper change		
	Baby play and providing toys		
	Dental health of babies		
	Weaning food		
Providing Information	Vaccination and health examination		

16-3. Help needed from spouses/partners

Service item	Detailed description of service item	Yes	No
Education for husbands or partners	Husbands' baby care (e.g., diaper changing)		
	Baby bath by partners		
	Massage for the mother		
	Encouraging the mother		
	Emotional support for the depressed mother		
	Contraception		

16-4. Help needed regarding the home environment

Service item	Detailed description of service item	Yes	No
Parenting and home environment	Housing issues		
	Financial issues		
	Receiving information about the home environment		
	Assessing and improving safety risk factors at home		
	Infant and childcare services in public health centers		
	Relationship with older siblings		
	Relationship with extended family members		
	Health issues of siblings or partner		
	Addressing health emergencies		
	Use of a car seat for infants		

16-5. Please describe services other than those listed above that you need until your child reaches 24 months of age.

[Common items for pregnant women and mothers with children aged less than 24 months]

Following questions ask about sensitive information regarding your financial status and lifestyle. If you answer these questions, we will use the valuable information to create a more detailed and realistic service. Your answers are protected by the Statistics Act, cannot be linked to a specific individual, and will not be used for any other purposes than reporting of the results.

DQ1. What is your yearly household income?

□ Less than 10,000,000 KRW

□ 10,000,000 to 20,000,000 KRW

□ 20,000,000 to 30,000,000 KRW

□ 30,000,000 to 50,000,000 KRW

□ 50,000,000 to 70,000,000 KRW

□ 70,000,000 to 100,000,000 KRW

□ More than 100,000,000 KRW

DQ2. What is the highest education level that you completed?

□ Below high school

□ College

□ University or university graduation

□ Graduate school

DQ3. Do you drink more than twice a week?

□ Yes □ No

DQ4. Do you currently smoke cigarettes (including e-cigarettes)?

□ I have never smoked cigarettes.

□ I currently smoke cigarettes.

□ I used to smoke cigarettes but have quit.

DQ5. Where did you mainly live and grow up?

□ I was born in South Korea and live in South Korea.

□ I was born abroad and live in South Korea.

□ I am a foreigner who was born abroad and lives in South Korea.

□ I was born in South Korea and live in South Korea, but I have lived abroad.

□ I am a South Korean citizen but mainly live abroad. I sometimes live in South Korea.
