# Supplementary material for: Is a universal nurse home visiting program possible? A cross-sectional survey of nurse home visitation service needs among pregnant women and mothers with young children
Source: PLoS One. 2022 Aug 4;17(8):e0272227. doi: 10.1371/journal.pone.0272227 (PMC9352077; doi:10.1371/journal.pone.0272227)
Supplement: S2 Fig — (PDF) [file pone.0272227.s002.pdf]

S2 Fig. Questionnaire for the survey of nurse home visitation service needs among pregnant women and mothers with children below the age of 24 months (Korean version)

임신 여성 및 만 24개월 이하의 자녀를 둔 어머니의 가정방문 프로그램 요구도 조사 설문지

[설문조사를 위한 점검문항]

SQ1. 귀하의 성별은 어떻게 되십니까? 1) 남성 2) 여성

SQ2. 귀하의 연령은 어떻게 되십니까? 출생연도 : ( )년

SQ3. 귀하의 거주지역은 어떻게 되십니까? (지도에서 광역시/도를 선택하시면 됩니다)

SQ4. 귀하께서는 현재 자녀가 몇 명이십니까? (임신 중 태아 포함)

1) 5명 2) 4명 3) 3명 4) 2명 5) 1명 6) 0명

SQ4-1. 귀하께서는 현재 임신중이십니까?

1) 예 2) 아니오

SQ4-2. 귀하의 자녀 연령은 어떻게 되십니까?

첫째자녀 : [ ]년 [ ]개월 둘째자녀 : [ ]년 [ ]개월

셋째자녀 : [ ]년 [ ]개월 넷째자녀 : [ ]년 [ ]개월

다섯째자녀 : [ ]년 [ ]개월

안녕하십니까?

이 설문조사는 '산전·조기 아동기 방문건강관리 방안 연구'(책임연구원: 서울대학교 의과대학 강영호 교수)의 일환으로 진행됩니다.

설문결과는 통계적으로 분석되어 엄마와 아기가 필요로 하는 간호사의 가정방문 서비스 개발에 활용됩니다. 본 설문조사에 참여하지 않거나 중간에 그만두시는 것으로 인하여 어떠한 불이익도 발생하지 않음을 알려드립니다.

설문에 소요되는 예상시간은 10분~20분으로 설문조사와 관련하여 질문이 있으실 경우 하단의 문의하기 버튼을 활용해주시기 바랍니다.

개인별 응답결과는 공개되지 않으며, 모든 개인정보는 보호됩니다. 이 조사는 자발적으로 참여의사가 있으신 분에 한하여 진행됩니다.

○ 조사 계속 진행　　○ 조사 진행 거절

[임신 여성 및 만 24개월 이하 자녀를 둔 어머니 공통 문항]

1. 임신 여성에게는 출산 준비를 돕고, 아기를 낳은 여성에게는 출산 후 건강, 아기돌보기, 모유수유를 도와주며, 아기의 성장발달을 확인하고 상담 활동을 하는 보건소 간호사가 무료로 여러분 댁에 직접 방문하여 서비스하는 것에 대해 의견은 어떠십니까?

○ 간호사가 우리 집을 방문하는 것에 대해 찬성한다.

○ 간호사가 우리 집을 방문하는 것에 대해 반대한다.

2. 보건소 간호사가 여러분의 댁으로 직접 방문하여 위와 같은 가정방문 프로그램을 무료로 제공한다면 서비스를 받으실 의향이 있으십니까?

○ 전혀 받고 싶지 않다.

○ 받고 싶지 않다.

○ 보통이다.

○ 받고 싶다.

○ 매우 받고 싶다.

지난 일주일 동안의 기분에 대하여 답하여 주십시오.

3. 나는 잘 웃고 주변 일들의 재미난 면을 잘 볼 수 있었습니다.

□ 예전과 마찬가지로 그러하였습니다.

□ 예전보다는 조금 덜 그러하였습니다.

□ 예전보다 확실히 많이 그러하지 못하였습니다.

□ 전혀 그렇지 못하였습니다.

4. 나는 즐거운 마음으로 미래에 일어날 일들을 기대하였습니다.

□ 예전과 마찬가지로 그러하였습니다.

□ 예전보다는 조금 덜 그러하였습니다.

□ 예전보다는 확실히 덜 그러하였습니다.

□ 거의 그러하지 못하였습니다.

5. 일이 잘못될 경우 나는 지나치게 나 스스로를 탓하였습니다.

□ 예, 대부분의 경우(대체로) 그러하였습니다.

□ 예, 종종 그러하였습니다.

□ 자주 그렇지는 않았습니다.

□ 아니요, 전혀 그렇지 않았습니다.

6. 나는 특별한 이유 없이 초조하고 불안하였습니다.

□ 아니요, 전혀 그렇지 않았습니다.
□ 거의 그렇지 않았습니다.

□ 예, 때때로 그러하였습니다.
□ 예, 자주 그러하였습니다.

7. 나는 뚜렷한 이유 없이 두려움 혹은 공포심을 느꼈습니다.

□ 예, 꽤 자주 그러하였습니다.
□ 예, 종종 그러하였습니다.

□ 아니요, 그다지 그렇지 않았습니다.
□ 아니요, 전혀 그렇지 않았습니다.

8. 상황이 내게는 너무 버겁게 느껴졌습니다.

□ 예, 대부분의 경우 상황을 전혀 감당할 수 없었습니다.

□ 예, 예전처럼 상황을 처리하지 못하는 때가 종종 있었습니다.

□ 아니요, 대부분의 경우 상황을 잘 처리할 수 있었습니다.

□ 아니요, 늘 그렇듯이 상황을 잘 처리했습니다.

9. 나는 너무 불행해서 잠을 이루기가 어려웠습니다.

□ 예, 대부분의 경우 그러하였습니다.
□ 예, 종종 그러하였습니다.

□ 아니요, 자주 그렇지는 않았습니다.
□ 아니요, 전혀 그렇지 않았습니다.

10. 나는 슬프고 비참하다고 느꼈습니다.

□ 예, 대부분의 경우 그러하였습니다.
□ 예, 꽤 자주 그러하였습니다.

□ 아니요, 자주 그렇지는 않았습니다.
□ 아니요, 전혀 그렇지 않았습니다.

11. 너무 불행하다고 느껴서 울었습니다.

□ 예, 대부분의 경우 그러하였습니다. □ 예, 꽤 자주 그러하였습니다.

□ 아주 가끔 그러하였습니다. □ 아니요, 전혀 그렇지 않았습니다.

12. 자해하고 싶다는 생각이 들었습니다.

□ 예, 꽤 자주 그러하였습니다. □ 때때로 그러하였습니다.

□ 거의 그렇지 않았습니다. □ 전혀 그렇지 않았습니다.

임신 중에 간호사의 가정방문을 받으신다면, 어떤 도움을 원하십니까? 해당 항목에 대해 도움을 받기 원하시면 '예', 원하지 않으면 '아니오'로 답해 주시기 바랍니다. 현재 임신 중이신 분은 현재 필요한 도움을 생각하여 답을 해주시고, 현재 아기를 키우시는 분은 임신하였을 때를 생각해서 답을 해주시기 바랍니다.

13-1. 엄마 자신에게 필요한 도움

필요한 도움	구체적인 도움 내용	예	아니오
출산준비	분만시 진통 / 분만 진행과정 알기/<br>분만시 힘주기와 힘빼기 방법/ 호흡법		
	엄마되기 위한 준비하기		
	임신 초·중·후기 임부와 태아의 변화		
	출산 징후(신호) 알기		
자가 간호	임신 중 영양/운동에 대해 알고 실천하기		
	임신 중 변비 해결하기		
	임신 중 회음부 간호법 알기		
	임신 중 구강관리		
심리적 지지	임신 중 감정변화 조절 이해하기		
	스트레스 관리하기		
	우울증 검사하기		
아기와<br>상호작용	태교		
수유준비	수유에 대한 전반적인 설명을 듣고 배우기		
	모유수유 준비하기		

13-2. 출산 후 아기를 돌보는 데 필요한 도움

필요한 도움	구체적인 도움 내용	예	아니오
성장발달	신생아 성장발달 알기		

아기상태파악	영아돌연사증후군 예방		
	신생아 정상 체온 알기		
	배꼽과 피부상태 확인하기		
	소변·대변 상태 및 횟수 확인하기		
	신생아 이상 증상(설사, 구토, 발열) 파악		
아기 돌보기	베이비 마사지		
	아기의 수면 이해하기		
	목욕시키는 법 배우기		
	울음 달래는 법 이해하기		
	기저귀 갈아주는 법 배우기		
	체온 확인		
	피부발진 여부 확인		
	수유량		

13-3. 배우자(혹은 파트너)에게 필요한 도움

필요한 도움	구체적인 도움 내용	예	아니오
배우자 혹은 파트너 교육	임신과 분만 과정 이해하기		
	임신과 분만 과정 지지해 주기 / 진통 시 배우자(혹은 파트너)가 할 수 있는 긴장 이완 방법(호흡법, 마사지)		
	신생아 돌보는 방법 배우기		
	피임		

13-4. 양육환경에 대해서 받고 싶은 도움

필요한 도움	구체적인 도움 내용	예	아니오
양육환경	실내온도 맞추기		
	가정 내 안전 위험요인 확인하고 개선하기		
	양육환경 준비에 필요한 정보제공		

13-5. 앞의 내용 외에, 임신 중인 현재나 임신 당시에 꼭 필요했던 서비스를 말씀해주세요.

[생후~만 24개월 이하 자녀를 둔 어머니]

설문조사는 가장 최근에 출산한 자녀를 대상으로 작성해주시기를 부탁드리겠습니다. 감사합니다.

지난 2-3주 동안 본인이 어떻게 느껴왔는지 체크해주세요.

1. (지난 2-3주 동안) 나는 내 아이를 돌보는 것에 대해 자신감을 느꼈다.

○ 예. 대부분 또는 모든 시간 동안 그러하였다.

○ 예. 어느 정도 시간 동안 그러하였다.

○ 아니오. 별로 그러하지 않았다.

○ 아니오. 전혀 또는 거의 그러하지 않았다.

2. (지난 2-3주 동안) 임신하기 전에 누렸던 삶을 놓쳤다.

○ 아니오. 전혀 또는 거의 그러하지 않았다.

○ 아니오. 별로 그러하지 않았다.

○ 예. 어느 정도 시간 동안 그러하였다.

○ 예. 대부분 또는 모든 시간 동안 그러하였다.

3. (지난 2-3주 동안) 나는 내 아이가 울 때 대처하기 어렵다고 느꼈다.

○ 아니오. 전혀 또는 거의 그러하지 않았다.

○ 아니오. 별로 그러하지 않았다.

○ 예. 어느 정도 시간 동안 그러하였다.

○ 예. 대부분 또는 모든 시간 동안 그러하였다.

4. (지난 2-3주 동안) 나는 내 아이와 친근하다고 느꼈다.

○ 예. 대부분 또는 모든 시간 동안 그러하였다.

○ 예. 어느 정도 시간 동안 그러하였다.

○ 아니오. 별로 그러하지 않았다.

○ 아니오. 전혀 또는 거의 그러하지 않았다.

5. (지난 2-3주 동안) 나는 외롭거나 홀로 된 것처럼 느꼈다.

○ 아니오. 전혀 또는 거의 그러하지 않았다.

○ 아니오. 별로 그러하지 않았다.

○ 예. 어느 정도 시간 동안 그러하였다.

○ 예. 대부분 또는 모든 시간 동안 그러하였다.

6. (지난 2-3주 동안) 나는 삶이 지겹다고 느꼈다.

○ 아니오. 전혀 또는 거의 그러하지 않았다.

○ 아니오. 별로 그러하지 않았다.

○ 예. 어느 정도 시간 동안 그러하였다.

○ 예. 대부분 또는 모든 시간 동안 그러하였다.

7. (지난 2-3주 동안) 나는 주변으로부터 도움을 받지 못하고 있다고 느꼈다.

○ 아니오. 전혀 또는 거의 그러하지 않았다.

○ 아니오. 별로 그러하지 않았다.

○ 예. 어느 정도 시간 동안 그러하였다.

○ 예. 대부분 또는 모든 시간 동안 그러하였다.

8. (지난 2-3주 동안) 내가 필요할 때 다른 사람의 도움이나 조언을 구하는 데에 어려움이 없다고 느꼈다.

○ 예. 대부분 또는 모든 시간 동안 그러하였다.

○ 예. 어느 정도 시간 동안 그러하였다.

○ 아니오. 별로 그러하지 않았다.

○ 아니오. 전혀 또는 거의 그러하지 않았다.

9. (지난 2-3주 동안) 내 아이를 대할 때에 신경이 곤두서거나 편안하지 않다고 느꼈다.

○ 아니오. 전혀 또는 거의 그러하지 않았다.

○ 아니오. 별로 그러하지 않았다.

○ 예. 어느 정도 시간 동안 그러하였다.

○ 예. 대부분 또는 모든 시간 동안 그러하였다.

10. (지난 2-3주 동안) 내 아이에게 무슨 일이 일어날까봐 걱정을 했다.

○ 아니오. 전혀 또는 거의 그러하지 않았다.

○ 아니오. 별로 그러하지 않았다.

○ 예. 어느 정도 시간 동안 그러하였다.

○ 예. 대부분 또는 모든 시간 동안 그러하였다.

11. (지난 2-3주 동안) 나는 내 아이 때문에 화가 나거나 짜증이 났다.

○ 아니오. 전혀 또는 거의 그러하지 않았다.

○ 아니오. 별로 그러하지 않았다.

○ 예. 어느 정도 시간 동안 그러하였다.

○ 예. 대부분 또는 모든 시간 동안 그러하였다.

12. (지난 2-3주 동안) 다른 엄마들만큼 좋은 엄마가 아닐까봐 걱정을 하고 있다.

○ 아니오. 전혀 또는 거의 그러하지 않았다.

○ 아니오. 별로 그러하지 않았다.

○ 예. 어느 정도 시간 동안 그러하였다.

○ 예. 대부분 또는 모든 시간 동안 그러하였다.

13. (지난 2-3주 동안) 나는 죄책감을 느꼈다.

○ 아니오. 전혀 또는 거의 그러하지 않았다.

○ 아니오. 별로 그러하지 않았다.

○ 예. 어느 정도 시간 동안 그러하였다.

○ 예. 대부분 또는 모든 시간 동안 그러하였다.

14. 육아를 하면서 가장 힘들었던 시기는 언제였습니까?

○ 신생아기(0~4주)

○ 2~3개월(5~12주)

○ 4~6개월(13~24주)

○ 7~12개월

○ 13~24개월

15. 힘들었던 이유는 무엇입니까 (복수응답 가능)?

□ 신체적 피로에 따른 고단함

□ 모유수유의 어려움

□ 아기가 무엇을 원하는지 알기 어려움

□ 아기의 울음을 달래기 어려움

□ 우울감

□ 고립감

□ 배우자 또는 파트너의 도움을 받지 못하여

□ 주변의 도움을 받지 못하여

□ 경제적인 어려움

□ 경력 단절에 대한 걱정

□ 기타(   )

다음은 출산 후 아이의 연령 만24개월 이내에 간호사가 가정을 방문하여 도움을 드릴 수 있는 내용들입니다. 이 시기에 간호사의 가정방문을 받으신다면, 어떤 도움을 원하십니까? 해당 항목에 대해 도움을 받기 원하시면 '예', 원하지 않으면 '아니오'로 답해 주시기 바랍니다.

16-1. 엄마 자신에게 필요한 도움

필요한 도움	구체적인 도움 내용	예	아니오
수유	수유에 대한 전반적인 설명을 듣고 배우기		
	모유수유, 분유수유, 혼합 수유의 구체적인 방법		
	수유량과 횟수에 대해 알기		
	수유 후 트림시키기		
신체 간호	유방 마사지하기		
	유선염 예방과 치료하기		
	요실금 간호하기		

	체중관리하는 방법 배우기		
	산후체조(스트레칭 등)		
자가 간호	영양/신체적 활동에 대해 알고 실천하기		
	오로 확인하고 대처하기		
	피임		
육아기술	아기에게 반응하는 방법 알기		
	훈육에 대해 알기		
심리적 지지	불안에 대해 이야기하고 안심하기		
	격려와 지지 받기		
	산후 우울 검사 받기		
	직업과 경력에 대한 상담받기		
양육신념	엄마 자신의 미래에 대한 포부		
	아기의 미래에 대한 포부		

16-2. 아기를 돌보는 데 필요한 도움

필요한 도움	구체적인 도움 내용	예	아니오
아기상태파악	발육상태 판단하기		
	영아돌연사증후군		
	아기 몸 관찰하기		
	배꼽과 피부상태 확인하기		
	소변·대변 상태 및 횟수 확인하기		
아기 돌보기	아기와 상호작용하기		
	베이비 마사지하기		
	목욕시키기		
	울음 달래기		
	잠재우기		
	손톱발톱 깎아주기		
	기저귀 갈기		
	월령별 놀이, 장난감 활용		
	잇몸 마사지, 구강 내 청결		
	이유식 먹이기		
정보제공	예방접종, 영유아 건강검진 정보제공 받기		

16-3. 배우자(혹은 파트너)에게 필요한 도움

필요한 도움	구체적인 도움 내용	예	아니오
배우자 혹은	아기돌보기 기술 배우기 / 신생아 안기, 목욕, 트림, 기저귀 갈기 등		

파트너 교육	목욕시키는 방법 배우기		
	아내 마사지 해주기		
	아내 격려하기		
	아내의 산후 우울 시 정서적 지지		
	피임		

16-4. 양육환경에 대해서 받고 싶은 도움

필요한 도움	구체적인 도움 내용	예	아니오
양육환경	주거문제		
	경제적 문제		
	양육환경에 대한 정보받기		
	가정내 안전 위험요인 확인하고 개선하기		
	보건소 영유아 서비스 정보 제공받기		
	큰 아이와의 관계 맺기에 대해 배우기		
	확대가족(친정, 시댁 등)과의 관계 맺기		
	다른 가족들(큰 아이, 배우자 등)의 건강		
	응급상황에 따른 대처방법		
	카시트 사용방법		

16-5. 위의 내용 외에, 아기가 태어난 후 만 두 돌까지 꼭 필요했던 서비스를 말씀해주세요.

[임신 여성 및 만 24개월 이하 자녀를 둔 어머니 공통 문항]

이어지는 질문은 가정형편과 생활습관에 대한 민감한 내용을 포함하고 있습니다. 그러나 질문에 응답해주신다면 좀 더 구체적이고 현실적인 서비스를 만드는 데 귀중한 자료로 사용하겠습니다. 응답하신 내용은 통계법에 의해 보호가 되고 개인을 특정할 수 없으며 결과 보고 목적 외에 사용하지 않습니다.

DQ1. 귀댁의 연간 가구 소득 수준은 어떻게 되십니까?

○ 1,000만원 미만
○ 1,000만원~2,000만원 미만
○ 2,000만원~3,000만원 미만
○ 3,000만원~ 5,000만원 미만
○ 5,000만원~ 7,000만원 미만
○ 7,000만원 ~ 1억 미만
○ 1억 이상

DQ2. 귀하의 최종 학력은 어떻게 되십니까?

○ 고등학교 졸업 이하 ○ 전문대학 ○ 대학 중퇴 또는 졸업 ○ 대학원

DQ3. 귀하는 현재 주 2회 이상 음주를 하시나요?

○ 예 ○ 아니오

DQ4. 귀하는 현재 담배(전자담배도 포함)를 피우십니까?

○ 담배 피운 경험이 없다.
○ 현재 담배를 피우고 있다.
○ 담배를 피우다 현재는 끊은 상태이다.

DQ5. 귀하가 주로 자라고 살아온 곳이 어디 입니까?

○ 대한민국에서 태어났으며 현재도 살고 있다.
○ 외국에서 태어나서 현재 대한민국에서 살고 있다.
○ 외국에서 태어나서 현재 대한민국에서 살고 있는 외국인이다.
○ 대한민국에서 태어나서 주로 대한민국에서 살고 있으나 외국에서 살았었다.
○ 대한민국 국적이나 주로 외국에서 살고 있으며 가끔 대한민국에서 거주 하고 있다.
