# Supplementary material for: Is a universal nurse home visiting program possible? A cross-sectional survey of nurse home visitation service needs among pregnant women and mothers with young children
Source: PLoS One. 2022 Aug 4;17(8):e0272227. doi: 10.1371/journal.pone.0272227 (PMC9352077; doi:10.1371/journal.pone.0272227)
Supplement: S1 Table — (PDF) [file pone.0272227.s003.pdf]

## S3 Table. Intention to use nurse home visitation services among pregnant women and mothers with children below the age of 24 months in Seoul and other regions of Korea

| | Seoul | | Other regions | |
|---|---|---|---|---|
| | N (%) | Intention to use home visitation services, Willing or very willing (%) | N (%) | Intention to use home visitation services, Willing or very willing (%) |
| Total study participants | 92 (100.0) | 80 (87.0) | 408 (100.0) | 326 (79.9) |
| Pregnancy status | | | | |
| Pregnant women | 28 (30.4) | 22 (78.6) | 122 (29.9) | 102 (83.6) |
| Women with children below the age of 24 months | 64 (69.6) | 58 (90.6) | 286 (70.1) | 224 (78.3) |
| Women's age (years) | | | | |
| 20-29 | 5 (5.4) | 5 (100.0) | 53 (13.0) | 44 (83.0) |
| 30-39 | 79 (85.9) | 69 (87.3) | 336 (82.4) | 269 (80.1) |
| 40-49 | 8 (8.7) | 6 (75.0) | 19 (4.7) | 13 (68.4) |
| No. of children (including fetus) | | | | |
| 1 | 52 (56.5) | 48 (92.3) | 245 (60.0) | 198 (80.8) |
| 2 | 34 (37.0) | 28 (82.4) | 136 (33.3) | 109 (80.1) |
| 3 or more | 6 (6.5) | 4 (66.7) | 27 (6.5) | 19 (70.4) |
| Annual household income (USD) | | | | |
| Less than 20,000 | 2 (2.2) | 2 (100.0) | 18 (4.4) | 11 (61.1) |
| 20,000-29,999 | 5 (5.4) | 4 (80.0) | 43 (10.5) | 32 (74.4) |
| 30,000-49,999 | 22 (23.9) | 21 (95.5) | 149 (36.5) | 124 (83.2) |
| 50,000-69,999 | 32 (34.8) | 26 (81.3) | 122 (29.9) | 97 (79.5) |
| 70,000-999,999 | 22 (23.9) | 18 (81.8) | 64 (15.7) | 50 (78.1) |
| 100,000 or over | 9 (9.8) | 9 (100.0) | 12 (2.9) | 12 (100.0) |
| Educational attainment | | | | |
| High school or less | 4 (4.3) | 4 (100.0) | 38 (9.3) | 28 (73.7) |
| College | 9 (9.8) | 6 (66.7) | 84 (20.6) | 60 (71.4) |
| University or more | 79 (85.9) | 70 (88.6) | 286 (70.1) | 238 (83.2) |
| Alcohol drinking, 2+ times per week | | | | |
| Yes | 13 (14.1) | 11 (84.6) | 66 (16.2) | 48 (72.7) |
| No | 79 (85.9) | 69 (87.3) | 342 (83.8) | 278 (81.3) |
| Cigarette smoking | | | | |
| No experience of smoking | 74 (80.4) | 65 (87.8) | 335 (82.1) | 268 (80.0) |
| Currently smoking | 2 (2.2) | 1 (50.0) | 10 (2.5) | 7 (70.0) |
| Ex-smokers | 16 (17.4) | 14 (87.5) | 63 (15.4) | 51 (81.0) |

| | | | | |
|---|---|---|---|---|
| Edinburgh Depression Scale | | | | |
| Less than 13 | 73 (79.3) | 64 (87.7) | 294 (72.1) | 235 (79.9) |
| 13 or over | 19 (20.7) | 16 (84.2) | 114 (27.9) | 91 (79.8) |
